# Size Matters — Determinants of Modern, Community-Oriented Mental Health Services

**DOI:** 10.3390/ijerph110808456

**Published:** 2014-08-19

**Authors:** Taina Ala-Nikkola, Sami Pirkola, Raija Kontio, Grigori Joffe, Maiju Pankakoski, Maili Malin, Minna Sadeniemi, Minna Kaila, Kristian Wahlbeck

**Affiliations:** 1 Department of Psychiatry, University Hospital Region, Hospital District of Helsinki and Uusimaa, Välskärinkatu 12, FI-00029 HUS, Finland; E-Mails: sami.pirkola@hus.fi (S.P.); grigori.joffe@hus.fi (G.J.); 2 Department of Mental Health and Substance Abuse Service, National Institute for Health and Welfare, Mannerheimintie 170, FI-00270 Helsinki, Finland; E-Mails: maiju.pankakoski@thl.fi (M.P.); maili.malin@thl.fi (M.M.); minna.sadeniemi@thl.fi (M.S.); kristian.wahlbeck@thl.fi (K.W.); 3 Hjelt Institute, Medical Faculty, University of Helsinki, FI-000014 Helsinki, Finland; E-Mail: minna.kaila@helsinki.fi; 4 School of Health Sciences, University of Tampere, Medisiinarinkatu 3, FI-33014 Tampere, Finland; E-Mail: sami.pirkola@uta.fi; 5 Department of Psychiatry, Hyvinkää Hospital Region, Hospital District of Helsinki and Uusimaa, Vanha Valtatie 198, FI-04500 Kellokoski, Finland; E-Mail: raija.kontio@hus.fi; 6 Department of Psychiatry, Porvoo Health Care Area, Hospital District of Helsinki and Uusimaa, Kaivokatu 37, FI-06100 Porvoo, Finland; E-Mail: minna.sadeniemi@hus.fi

**Keywords:** mental health services, community mental health services, catchment area

## Abstract

Governances, structures and contents of mental health services are being reformed across countries. There is a need for data to support those changes. The aim of this study was to explore the quality, *i.e*., diversity and community orientation, and quantity, *i.e*., personnel resources, of mental health and substance abuse services (MHS) and evaluate correlation between population needs and quality and quantity of MHS. The European Service Mapping Schedule—Revised (ESMS-R) was used to classify mental health and substance abuse services in southern Finland. Municipal-level aggregate data, local data on unemployment rate, length of education, age of retirement, proportion of single households, alcohol sales and a composite mental health index were used as indicators of population mental health needs. Population size correlated strongly with service diversity, explaining 84% of the variance. Personnel resources did not associate with diversity or community orientation. The indicators of mental health services need did not have the expected association with quality and quantity of services. In terms of service organization, the results may support larger population bases, at least 150,000 adult inhabitants, when aiming for higher diversity.

## 1. Introduction

Mental health and substance abuse services (MHS, including mental health and substance abuse services, regardless of their integration or separation) are undergoing governance, structural and content reform in Finland as well as elsewhere.

In Finland, municipalities are responsible for arranging public health care and social services for their residents and governmental steering is limited. Municipal health care is basically funded by taxes. For specialized health care, including specialized mental health care, municipalities form hospital districts. Finland is divided into 20 hospital districts. The primary care health centres have a gatekeeper role, and access to elective specialized care is usually by referral from primary care. As a general rule, smaller municipalities rely more heavily on the hospital district for provision of specialized mental health services, while some bigger (such as Helsinki, the capital city) municipalities tend to provide specialized mental care in their own health care organizations [1].

The autonomy of municipalities in organizing public services leads to heterogeneity between municipalities, depending on geographical area and the stage of psychiatric service reform toward community-based care [2,3]. This kind of geographical heterogeneity in provision of MHS and its implications for access to adequate treatment has been previously reported by Rocha *et al.* [4], who noted that in Spain heterogeneity causes individual and regional inequalities in access to adequate treatment and available resources. 

The MHS reform in Finland aims at patient-centered community-based outpatient services, while further limiting the use of institutional hospital-based services [5,6]. This deinstitutionalization process; *i.e*., restructuring and downsizing of psychiatric inpatient care, has been successful, as deemed by a decrease in suicides immediately or within one year post discharge [7]. In fact, well developed community mental health services in the municipalities have been associated with a lower level of suicides [3]. Overall, there has been an increased life expectancy for people with schizophrenia, other psychoses, mood disorders and neurotic disorders [8,9], but not for people with substance use disorders [9,10]. Currently despite the policy aim to prioritize community care, most MHS resources in southern Finland are still invested in hospital and non-hospital residential services, and low threshold outpatient services are relatively scarce [11]. Overall, both international [12] and national [3,7] data support the advantages, quality and effectiveness of more community-oriented, and multifaceted service structure.

Current mental health policies prioritize MHS which: (1) are balanced regarding community- and hospital-based care; (2) are based on well-developed community MHS; (3) appreciate and emphasize mobility and flexibility and (4) are characterized by abundant differentiation and diversity of service types [12,13,14]. Country- and global-level data about MHS resources, policy and development indicate a high degree of variability of MHS systems, even in countries within the same income categories. MHS provision patterns and structures are dependent on specific local circumstances such as general health care, income level, population density and state of MHS policy development [4,15,16]. When evaluating mental health services, assessment of local services should take into account local needs and resources [17].

The European Service Mapping Schedule (ESMS) is an instrument designed to investigate MHS structures, describe their major characteristics, provision of services and resource allocation [18,19,20]. It aims to find key elements and benchmark references for better and more differentiated MHS development, which allows for systematic and standardized comparisons of MHS.

This Finnish study is part of the nine country European REFINEMENT (REsearch on FINancing systems’ Effect on the quality of MENTal health care) project. The overarching aim of the project is to look at the relationship between different models of health care financing systems, and the extent to which mental health services can meet the goals of high quality, equity, efficiency and better long-term health outcomes [21,22]. The Finnish subproject focuses on the performance of Finnish MHS utilizing analyses of care pathways [11].

In this study we set out to analyse data on MHS service provision structure and volume with aggregate municipal-level indicators and to explore associations between needs of services and service provision. The aims were to evaluate: (1) MHS quality, using two indicators of quality: diversity and community orientation; (2) MHS quantity, using allocated personnel resources and (3) correlation between population needs and quality and quantity of MHS.

## 2. Methods

### 2.1. The Study Area

The study area included three hospital districts, those of Helsinki and Uusimaa, Kymenlaakso and Etelä-Karjala, owned and governed by 56 municipalities, altogether in the southernmost part of Finland. The total population in the study area is 1.8 million people, with 1.4 million adults (aged ≥ 18 years). The study area consists of nine non-overlapping catchment areas: Länsi-Uusimaa (Area 1), Lohja (Area 2), Hyvinkää (Area 3), Porvoo (Area 4), Helsinki (Area 5), Jorvi (Area 6), Peijas (Area 7), Kymenlaakso (Area 8) and Etelä-Karjala (Area 9). All catchment areas have a general hospital. Psychiatric hospital care is to some extent integrated in the general hospitals, but many areas still have separate, free-standing psychiatric hospitals. The total adult population size varied within areas from approximately 45,000 to (Area 1) to 500,000 inhabitants (Area 5). The study area is rather representative of the whole of Finland, because it covers about 30 per cent of the total country population. However, the study area is much more densely populated (174 inhabitants per square kilometers) than the country in total (16 inhabitants per square kilometers). 

### 2.2. Data Collection

Data collection was performed with the revised European Service Mapping Schedule (ESMS-R) tool [23]. An earlier version of the ESMS has been used previously in Finland [3] and it has been evaluated as a valid instrument in a European context [17,24,25]. In ESMS-R, mental health services are classified into 89 different Main Types of Care (MTC). The MTC is the main descriptor of the generic care function (e.g., mobile team or acute hospital care), provided by a Basic Stable Input of Care (BSIC). In practice, BSIC is the organizational unit providing the MTC. The operational description of a BSIC is based on organization, staff, premises and target population [26]. Information for classifying the MHS was collected in 2011–2012 systematically by three researchers, who received special training for use of the ESMS-R. Coding reliability was supported by a standardized handbook [23] as well as a systematic mapping procedure, case-based mapping training and assessment of inter-rater reliability by vignettes. Data were collected using public data sources, as well as interviews with health and social care representatives of municipalities and private care providers.

The mapping covers all municipalities in the study area, and includes all services within the scope of the municipalities’ obligation to arrange adult population MHS in primary care, secondary care, tertiary care level, social and substance abuse services. Only services for adults with mental health problems and substance abuse problems were included in the mapping; *i.e*., services for general health problems were excluded. Primary health care employees specialized in adult mental health care, such as psychiatrists, psychologists and psychiatric nurses in municipal health centres, were included. 

**Figure 1:**
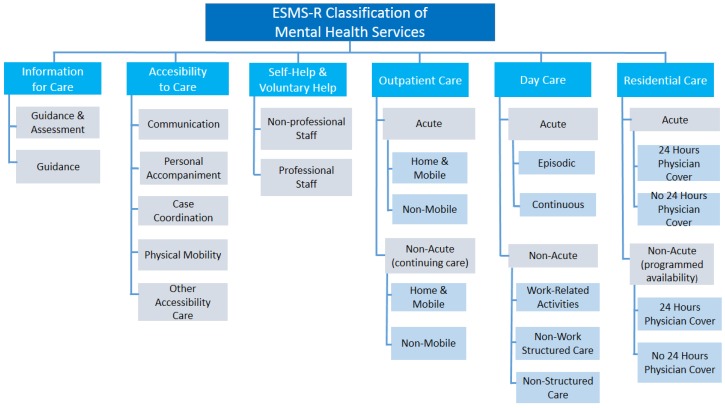
The European service mapping schedule (ESMS-R) mapping tree [23,26].

The mapped MTC were allocated to one of the six main branches of ESMS-R: (1) information for care (I); (2) accessibility to care (A); (3) self-help and voluntary help (S) (4) outpatient care (O); (5) day care (D) and (6) residential care (R) [23] (Figure 1).

### 2.3. Measures

#### 2.3.1. Diversity and Community Orientation

The count of different MTC codes was used to indicate the diversity of services, which was considered a quality indicator. The ratio of personnel in community-based services; *i.e*., outpatient and day care services *vs*. residential care, was used to indicate the community orientation, which was the second quality indicator used. The quantity of resources was expressed as amount of personnel in full-time equivalents (FTE) per 1000 inhabitants. The community orientation of MHS was evaluated by calculating a community-based service ratio by counting FTE of outpatient (O) and organized day care services (D) and dividing the count by the residential FTE. The self-help and voluntary (S) services were not included, as they did not have any FTE resources, only voluntary personnel. 

#### 2.3.2. Mental Health Needs

Several background variables were used to depict population mental health needs (Table 1). These are national and commonly used statistics that are supposed to indicate mental health needs. The Mental health index (MHI) was calculated for each catchment area using three years data on the number of suicides and suicide attempts, the number of persons eligible for special reimbursement for antipsychotic medication, and the number of persons on disability pension due to mental disorders (18–64 years old). The indicator describes through three dimensions the prevalence of mental health problems as a proportion to the population of the same age. Each of the three dimensions represents one third of the total weight of the disease group in the morbidity index. The MHI of Finland was given the value 100 and other areas or municipalities are compared with that baseline index. An MHI smaller than 100 indicate a better state of mental health than the average and a higher value indicates a worse state of mental health [27]. 

Adult population size was used because only adult MHS was mapped. The quantity and quality of MHS provision were expected to associate within MHI and other background variables as indicators of populations needs. Other background variables used were: years of education after primary school (education), average age of retirement, unemployment rate, ratio of single households and alcohol sales. These are established socioeconomic variables linked to mental health and were used to further explore differences in mental health needs between areas. Background information was collected from the Finnish Statistics and Indicator Bank “Sotkanet” using statistics data from 2011 [28]. 

### 2.4. Data Analysis

The nonparametric tests for independent samples (Mann Whitney Test) were used to test differences between areas due to non-normal distribution and a small number of units of analysis.

Spearman correlations were used to investigate the association between the different indicators of population needs and quality and quantity variables: the number of different MTC codes (diversity), total FTE per 1000 inhabitants (quantity) and community-based service FTE ratio (community orientation).

**Table 1:** Background variables of catchment areas *.

Catchment area	Länsi-Uusimaa	Lohja	Hyvinkää	Porvoo	Helsinki	Jorvi	Peijas	Kymen-laakso	Etelä-Karjala	Weighted Mean	SD	Finland
Area number	1	2	3	4	5	6	7	8	9			
Population (≥18 year)	35,316	70,192	138,973	74,079	497,814	227,605	185,984	141,085	107,612	164,295	138,626	4,202,852
Mental health index (not age adjusted)	82	84.5	72.1	73.5	83.9	65.9	78	110.8	104.7	83.4	13.1	100
Education **	3	3.2	3.5	3.3	4.1	4.6	3.4	3	3	3.4	0.6	3.4
Average age of retirement	60.2	59	58.6	59.3	59.3	58.9	58.9	58.4	58.8	59	0.5	58.7
Unemployment %	7.2	7.1	6	7.1	7.5	5.5	8	12.2	11.8	7.8	2.4	9.4
Single households (%)	40.1	34.8	34.2	35.2	49	34.4	38.1	43.9	43.6	41.5	5.3	41.2
Alcohol, sold (100%/ltr/inhabi-tant)	8.8	8	7.3	7.2	9.4	6.3	8.1	8.6	9.4	8.3	1	8.2

* Data Statistics Finland^©^ THL, SOTKAnet Statistics and Indicator Bank 2005–2013; ** Approximate education years (Scale 0–X) after primary school (in Finland approximaly 9 years education).

Scatterplots were used to explore and illustrate the associations between indicators of quality and quantity of services. Linear regression modelling was used to investigate the amount of variance in service quality and quantity explained by different indicators of population mental health need. Regression models were also adjusted for background variables, one by one (data not shown). The SPSS Statistics software version 21 was used for the analyses. 

## 3. Results

### 3.1. Characteristics of the Catchment Areas

There were differences between the catchment areas (Table 1). The mean MHI was 82.8 (SD 13.8), indicating that in the study area as a whole, mental health needs may be lower than the national average (100). In two areas; *i.e*., Kymenlaakso and Etelä-Karjala (8 and 9), MHI was higher (110.8 and 104.7) than the national average. Those areas encompass old paper industry cities, which have suffered from global and national economic turmoil, also indicated by their higher than average unemployment rate. Also higher than average unemployment rates were found in the same areas (8 and 9). A lower than national average (3.4) length of education was found in five areas. The proportion of single households was higher than mean in three areas. The highest alcohol sale was located in Helsinki capital city area (5) and in Etelä-Karjala area (9). Altogether, indicators of higher level of service needs seemed to cluster in Areas 8, 9 (Kymenlaakso and Etelä-Karjala) and to some extent in Area 5 (Helsinki).

### 3.2. Diversity

**Figure 2:**
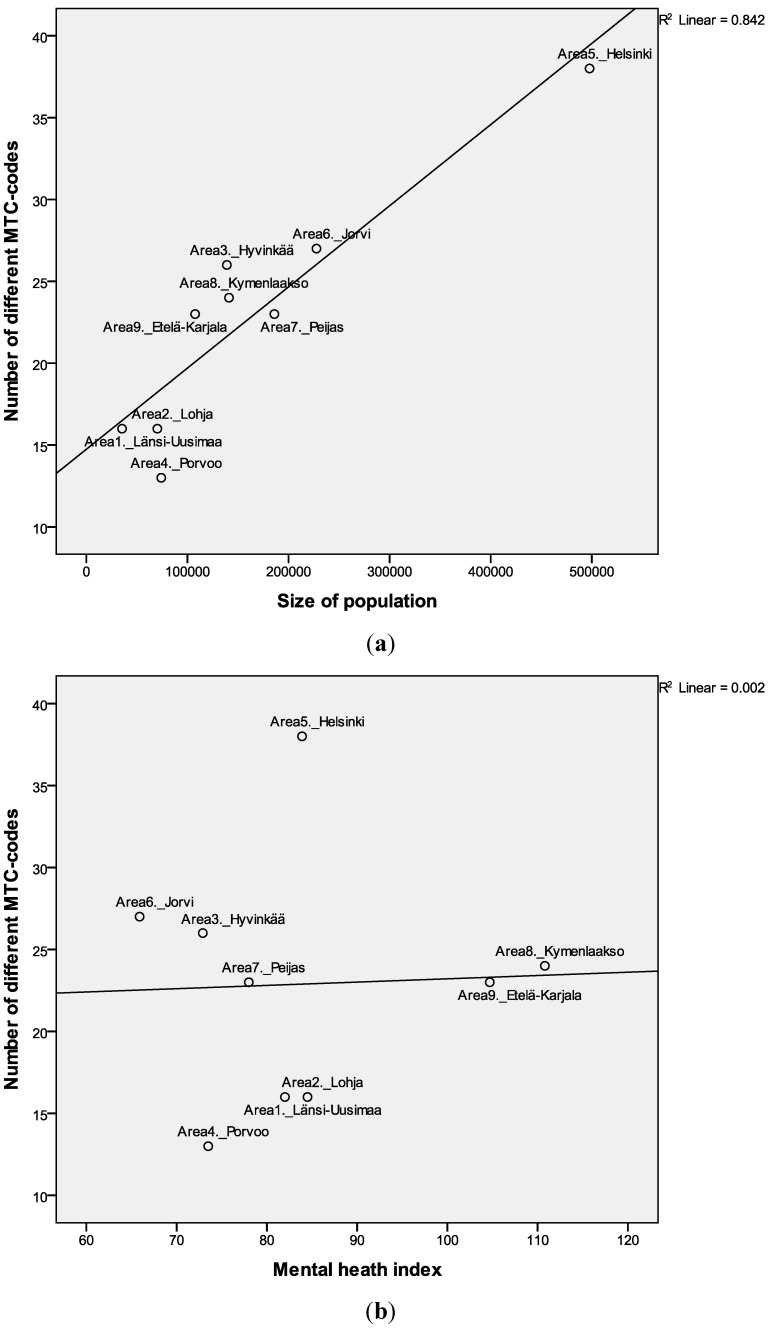
Diversity of services (MTC N = 89) association with size of population (**a**) and mental health index (**b**) (MHI national average 100). Linear regression lines and coefficients (r^2^) are shown.

Figure 2a,b present the scatterplots and regression lines between the diversity and size of adult population of the areas and mental health index. The size of population explained 84% of the variance in service diversity, shown by the regression coefficient r^2^ (Figure 2a). Table 2 summarizes the collected quantity and quality data of services; number of services (BSIC), allocated resources (FTE), number of different MTCs and community orientation indicators. 

The association between service diversity and population size of the catchment area was significant (r = 0.86, *p* = 0.003 (Table 3)). There was no statistically significant association between diversity and MHI.

Number of service units (BSIC) (N = 726) by main branches of ESMS-R varied widely related to size of areas (Table 2). Totally 56 different types of services (MTC) were recognized; *i.e*., 62% of all 89 possible types.

As an example, *Acute hospital care facilities (R3)* are unitswithout 24-hours physician cover in a registered hospital, and *Outpatient acute non-mobile health related care (O3.1)* provide specific care for a defined specific population group on a non-mobile basis [23]. 

The count of different services available in the catchment areas varied from 13 to 38 (mean 22.8, SD 7.5). There were no services mapped to accessibility to care (A) services which are more common in long-term disability services [26]. Information for care (I) services was found in five catchment areas corresponding to <0.5% of total FTE and 1.6% of (14/726) total number of units (BSIC). 

The different MTC count varied from 13 to 16 in the three smallest areas (less than 100,000 adults). Medium-sized catchment areas’ (100,000-150,000 adults) variation were 23–26 MTC. In areas with the largest population (180,000–500,000 adults) the range varied from 23 to 38 MTC.

**Table 2:** The number of different Main type of care (MTC) * by main branch of ESMS-R (diversity), resource allocation (FTE **) and community orientation of services.

Catchment area	Länsi-Uusimaa	Lohja	Hyvinkää	Porvoo	Helsinki	Jorvi	Peijas	Kymen-laakso		Etelä-Karjala		Total		Mean		SD
Area number	1	2	3	4	5	6	7	8		9						
**Quantity of services (Number of BSIC by ESMS-R main branches and resource allocation (FTE) **																
I Information for care	-	1	-	-	6	1	4	2		0		14		2.33		2.3
D Day care	6	3	10	7	29	9	10	16		16		106		11.8		7.7
S Self-help and voluntary care	4	9	24	9	29	15	11	23		18		142		15.8		8.3
O Outpatient care	6	11	19	12	55	19	22	31		19		194		21.6		14
R Residential care	7	16	41	15	72	25	30	44		20		270		30		20
Number of service units/BSIC	23	40	94	43	191	69	77	116		73		726		80.6		50
Day care (D) FTE	20.7	13	49.2	18.4	134.5	24.5	14	30		65.2		369		41.1		39
Outpatient care (O) FTE	44.6	49.3	131.9	48.2	563.9	198.7	173	128.3		90.11		1428		159		162
Residential care (R) FTE	113	179.2	396.7	130.2	983.0	252.9	283.5	397.4		176.5		2912		324		268
Total FTE	178.3	242.5	577.7	196.7	1701.4	479.1	477.5	557.7		331.7		4741		527		466
Total FTE/1000 adults	5.1	3.5	4.2	2.7	3.4	2.1	2.6	4.0		3.1		3.2		3.4		0.7
**Quality of services (Number of different MTC by ESMS-R main branches (diversity) and community orientation)**																
Number of different MTC	16	16	26	13	38	27	23	24		23		56		22.8		7.5
Community-based services =FTE D + O	65.3	62.3	181.1	66.58	420	223.2	187	158.3		155.3		1519		164		101
Community-based services =FTE D + O/1000 adult	1.85	0.89	1.3	0.9	0.84	0.98	1.01	1.12		1.44		1.03		1.13		0.3
Community orientation ratio ***	0.58	0.35	0.46	0.51	0.43	0.88	0.66	0.4		0.88		0.52		0.58		0.2

* European Service Mapping Schedule-Revised mapping tree’s main branch codes [23]: Accessibility (not found), Information for care, Day care, Self-help and voluntary care, Outpatient care and Residential care are main branches of Main types of Care (MTC). Totally 89 different MTC are possible on ESMS-R [23]; ** FTE, allocated full-time equivalents as personnel resources (located on units/ BSIC) on D, O and R services; *** Community orientation= community-based care (D+O) FTE/ residential care FTE ratio.

### 3.3. Community Orientation

**Figure 3:**
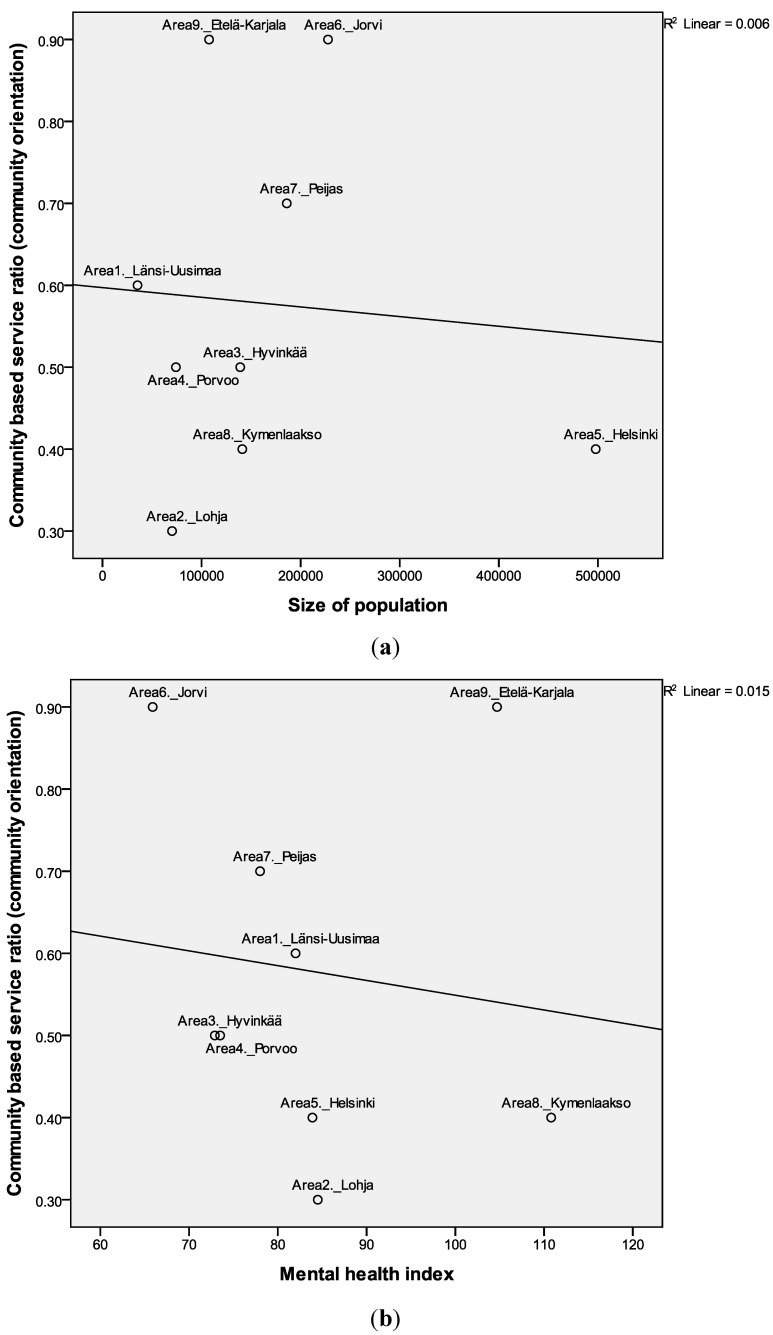
Community orientation (FTE allocated to community-oriented care *vs*. residential care) association with size of population (**a**) and mental health index (**b**) (MHI national average 100). Linear regression lines and coefficients (r^2^) are shown.

Ratio of mental health care staff allocated to community-based (O+D) services, related to the total FTE, was used as a quality indicator for community orientation (Table 2). There were no correlations between catchment areas’ size of population and personnel resources allocated on community-based services or community orientation (Table 3). Although the population sizes of areas were not influencing the community-based ratio, there were large differences between areas (Figure 3a,b). The study areas’ mean personnel allocation was 3.4 FTE per 1000 adult inhabitants (SD 0.9), and mean allocation to community-based services was 1.1 FTE per 1000 adult inhabitants (SD 0.3, range 0.8 to 1.8). Community-based orientation; *i.e*., ratio of mental health care staff allocated to community-based services, varied from 0.3 to 0.9 (mean 0.58, SD = 0.22). There were three areas with higher community orientation areas: Jorvi (6), Etelä-Karjala (9) and Peijas (7) (Mann Whitney U = 0, p = 0.019). The adult population size in these three areas varied from 100,000 to 230,000. Least community-based services were available in Lohja (2), Helsinki (5) and Kymenlaakso (8) areas.

**Table 3:** Correlations between sociodemographic variables, MTC-codes and resource allocation (FTE) (N = 9).

Variables		1	2	3	4	5	6	7	8	9	10
1	Population (+18years)	1									
2	Mental Health Index	−0.18	1.00								
3	Education	0.73 *	−0.67 *	1.00							
4	Unemployment	0.08	0.82 **	−0.56	1.00						
5	Age of retirement	−0.31	−0.19	−0.03	−0.25	1.00					
6	Single households	0.20	0.73 *	−0.38	0.85 **	0.12	1.00				
7	Alcohol sold	0.01	0.74 *	−0.44	0.77 *	0.09	0.85 **	1.00			
8	Different MTC-codes	0.86 **	−0.13	0.64	−0.05	−0.39	0.13	0.11	1.00		
9	Total FTE/1000 inhabitants	−0.50	0.35	−0.57	0.13	0.01	0.13	0.36	−0.11	1.00	
10	Community-based care FTE/1000 inhabitants	−0.40	0.09	−0.56	0.19	−0.31	−0.03	0.23	−0.13	0.45	1.00
11	Community orientation (Community/residential service FTE ratio)	0.13	−0.40	0.14	−0.06	−0.10	−0.16	−0.08	0.06	−0.49	0.50

* Correlation is significant at the 0.05 level—Spearman Correlation Test; ** Correlation is significant at the 0.01 level—Spearman Correlation Test.

### 3.4. Resource Allocation

**Figure 4:**
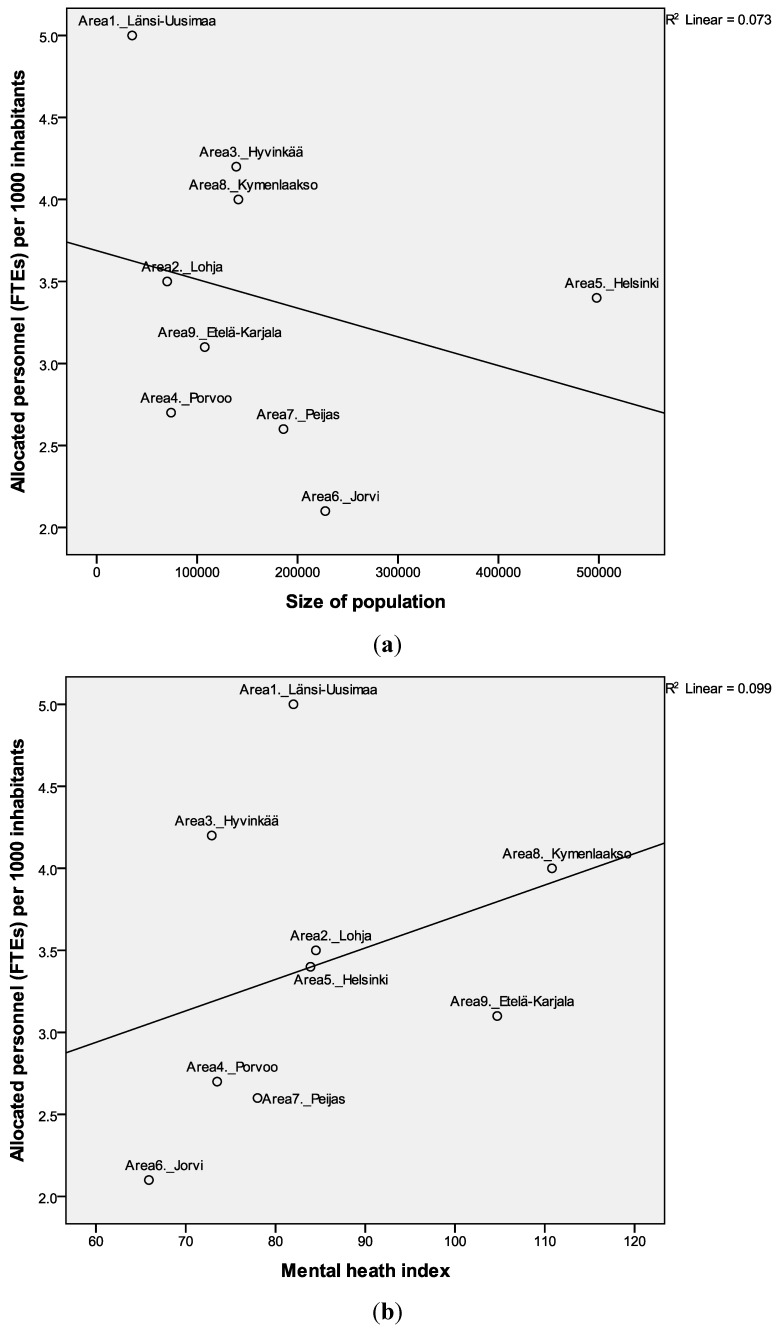
Allocated personnel resources FTE (Full time equivalents) per 1000 inhabitants associated with size of population (**a**) and mental health index (**b**) (MHI national average 100). Linear regression lines and coefficients (r^2^) are shown.

The second aim of the study was to evaluate the total resource allocation as an indicator of the quantity of MHS. The total numbers of allocated resources (FTE) by main branches of ESMS-R varied within the size of areas. A higher allocation of resources (measured as FTE/1000 adult inhabitants) did not associate with diversity (count of MTC, Figure 1c), size of population or MHI (Figure 4a,b). The areas varied regarding FTE per 1000 adult inhabitants from 2.1 to 5.1 (mean 3.4, SD 0.7) (Table 2).

The areas of Länsi-Uusimaa (1), Hyvinkää (3), Helsinki (5) and Kymenlaakso (8) had higher FTE per adult inhabitants than other areas (Mann Whitney U = 1.0, *p* = 0.027). These areas have secondary level MHS provided both by municipalities themselves and by hospital districts. 

### 3.5. Associations between Needs of Services and Service Provision

Many background variables that could indicate population mental health needs unsurprisingly correlated with the MHI. There were some positive significant correlations between MHI and unemployment (r = 0.82, *p* < 0.01), single households (r = 0.73, *p* < 0.05) and alcohol sold (r = 0.74, *p* < 0.05). The MHI was correlate negatively with education (r = −0.67, *p* < 0.05). However, MHI was not associated with the count of MTC codes (diversity), community-based ratio (community orientation) or total number of FTE per 1000 adult inhabitants (Figure 2b, Figure 3b and Figure 4b, Table 3). Thus there were not significant correlations between need and quality or quantity of services. The areas with high needs; *i.e*., Kymenlaakso (8) and Etelä-Karjala areas (9), did not have a higher resource allocation. 

## 4. Discussion

We investigated the diversity, community orientation and resource allocation of mental health and substance abuse services and their relation to population need indicators. We found that the only factor associated with a quality indicator, diversity of MHS, was the population size of the catchment area. It explained 84% of the variance in our sample of areas between 35,000–500,000 adults. The other background variables or population needs indicators did not associate with diversity. We were able to demonstrate a clear scale effect for MHS diversity, as measured by an available, standardized classification instrument [23,26]. This effect appears linear; *i.e*., there was no evidence for a ceiling effect in our sample of up to 500,000 adults. However, such an effect may be possible in samples greater than ours. Only two per cent of service diversity variation was explained by total staff resources per inhabitant. This indicates that even a high investment in number of staff does not create diversity in mental health service provision, if the population of the catchment area is insufficient.

The level of MHS community orientation, representing the stage of deinstitutionalization, was investigated using community-based service (*vs*. residential service) ratio (community orientation) as indicator. The personnel resource allocated to community-based services in catchment areas was on average less than half of the total FTE. No explanations for the large variation in community orientation were found, indicating that complex political, managerial and historical reasons play a role.

The quantity of services, defined as number of allocated personnel resources per 1000 adult inhabitants, did not correlate with need nor diversity or community orientation. Indicators of possibly worse health and service needs (MHI, unemployment and sold alcohol) did not seem to associate with higher resource allocation. This may indicate the complexity of need and services, and challenges in priorizing different needs. More research is needed in this regard. Notably, areas with separate, free-standing mental hospitals; Länsi-Uusimaa (Area 1), Lohja (Area 2) and Hyvinkää (Area 3), had a relatively low community orientation and relatively high personnel resource allocation. It is also notable that the Etelä-Karjala area (Area 9) has the highest integration of primary and secondary level MHS. We noticed that on three areas (1, 3, and 8) where the secondary level mental health services were provided both on municipality and hospital district level, allocated resources were higher than average. As this may not always be strategically planned, more integrated planning and coordination seems justified. 

The results suggest that the areas of at least 150,000 adult inhabitants and community service ratio more than 0.7 provides a richer diversity of services with less than average personnel resources. When staff resources per inhabitants increased this was associated with increased residential services. Staff resources for increased mobile-, acute-, intensive community-based services perhaps could be found by decreasing institutional services. Related to our findings this might mean that, shifting ten per cent of staff resources from residential services to community services (example Area 8 + 0.4 FTE) the total need of personnel may be substantially reduced (almost one FTE per 1000 adults).

### 4.1. Comparison to Previous Studies

Salvador-Carulla *et al*. [24] compared mental health systems in Italy and Spain and found great differences related to patterns of service provision and service use between countries. They suggested that developing innovative community services is linked to low hospital bed use, high rate of day services and contact to community. The ESMS was also used for describing mental health care outside of Europe [20] in a study of Spain and Chile urban areas. The results show partly similar gaps in MHS as in our study area. Based on the detailed data collected in our study, we can assume that both in Chile and our study area there is a relative lack of 24-hour mobile, non-mobile emergency psychiatric care and work-related services for persons with mental disorders [20]. When comparing our findings, in Chile there is a lack of non-acute residential services, whereas we found a lot of residential services, both acute and non-acute. 

Tibaldi *et al*. [29] recognized that areas with more intensive community services used, and less people living alone, had lower acute hospital bed occupancy rates. Those findings are supporting our results that areas with higher community orientation, and more diversity, need less total allocated resources. Earlier studies with the ESMS instrument have also reported that in rural areas with inadequate community-based services patients are more often admitted to hospital care [17]. Those findings support our findings from areas with lower population density (data not shown) and free-standing hospitals with a lower level of community orientation.

The development of mental health care systems in Western Europe is characterized by a common deinstitutionalization trend, including decreasing volume of inpatient treatment and improvement of community services. Becker *et al*. [30] reported findings from a number of studies describing mental health care in different European countries and comparing provision of care across countries. They found variability between national MHS, what results in different patterns of service use and service costs. We found similarly effected variability between study areas. Earlier Wilkinson *et al*. [31] constructed wider set of mental health indicators (over 80) from public data bases. The indicators they used were structured to: risk and protective factors and determinants, population health status, interventions, effectiveness of partnerships, service user experience and workforce capacity. They found out that service provision is, similarly with our findings inconsistent and does not always relate to the need, although inventories they used were fairly comprehensive [31]. 

We are aware of only few earlier studies on catchment area population size and quality of MHS. Kutash *et al*. [32] found that bigger population size is linearly linked to the level of implementation of mental health system of care approach. Wang *et al*. [33] found that smaller Californian counties (population less than 200,000) take a longer time to make decision for implementing a new staff-intensive evidence-based program. It should be noted, however, that the Californian healthcare distribution is in many ways very different from the Finnish one. In general it may be that when a large variation between or within areas exists, underlying local patterns like unemployment rates and numbers of single households need to be taken into account when research findings are discussed and implemented [24].

The MHS structure in the study areas did not seem to be based on population service needs. The observed differences in patterns of service provision may be due to the complexity of the organizing processes, too many independent organizations or lack of means for common strategic steering. Porter and Lee [34] suggested building integrated practice units with single administrative hubs and a large population base. This might help account for differences in need for services and integrate patient education, engagement and follow-up structures across health care systems on primary, secondary and tertiary level. This structure might also provide for increased diversity by enabling creation of specialized multidisciplinary teams with large population bases.

### 4.2. Strengths and Limitations

The strengths of this study include the use of the internationally validated instrument ESMS-R for classification and comparison of services. The strength of the ESMS-R is that the comparison includes the full coverage of MHS in the study area, including primary, secondary and tertiary care as well as social services and voluntary services. Data were collected by trained persons in strong cooperation with local stakeholders.

The limitations need to take account are linked strongly to need to understanding of national and local structure and patterns of MHS. Earlier comparisons between areas refer to effects of local history in the development of MHS. The differences in organizational structure, complexity of service networks and problems in definition of terms and classification have to be recognized when comparing services [26,31,35]. Data have been collected during a certain period, when changes in service organization have been going on. Therefore, the setting is considered cross-sectional and does not necessarily represent a later situation. This implicates a need for follow- up study with similar standardized methods.

The Mental Health Index (MHI) is a measure of endpoints of longstanding severe mental disorders, and processes that perhaps could have been prevented with more community-oriented services. Although it is currently used in national level planning, it may not be a particularly valid indicator of the whole spectrum of mental health needs. This may partly explain the fact, that we did not find a relationship between MHI and our quality indicators.

The ESMS-R instrument classifies mental health services according to main type of care provided. In cases where a variety of services is provided by each classifiable unit, additional services be overshadowed by the need to establish a main type of care, and thus ESMS-R may not always correctly reflect the ability to provide highly flexible services by a single unit. This may introduce a bias in the count of MTC, if units in smaller catchment areas more often provide more than one type of care. Individual units may also be able to differentiate and offer diversity of services, in case of well-trained teams.

## 5. Conclusions and Recommendations

This study demonstrates that the size of catchment area enables increased MHS diversity, which appears to be non-dependent on per capita personnel resources in the services. In our urban and semi-urban public service setting, large differences in community orientation and total allocated staff resources do not seem to be determined by service needs. A recommendation could be that areas with low community ratio continue to reallocate resources to community-oriented services and reduce residential care. Diversity, on the other hand, may possibly require large population bases—in this study up to at least 150,000 inhabitants, but possibly even up to 500,000. 
